# P-641. An Untimely Death: Mortality in Young Adults with Viral Syndromes including COVID-19, Influenza, and Respiratory Syncytial Virus

**DOI:** 10.1093/ofid/ofaf695.854

**Published:** 2026-01-11

**Authors:** Karlee Grudi, Claudine Jurkovitz, Lianteng Zhi, Denise Taylor, David Lynn, Marci Drees

**Affiliations:** ChristianaCare, Newark, Delaware; ChristianaCare Health Services, Inc., Wilmington, Delaware; Christiana care, Newark, Delaware; ChristianaCare, Newark, Delaware; iREACH, Wilmington, Delaware; ChristianaCare, Newark, Delaware

## Abstract

**Background:**

Older age is a well-known risk factor for mortality from respiratory viruses, including COVID-19, influenza, and respiratory syncytial virus (RSV). There is limited information on mortality risk in younger patients with these diseases. The aim of this study was to identify comorbidities and risk factors associated with virus-related death in young adult patients.

**Figure:**
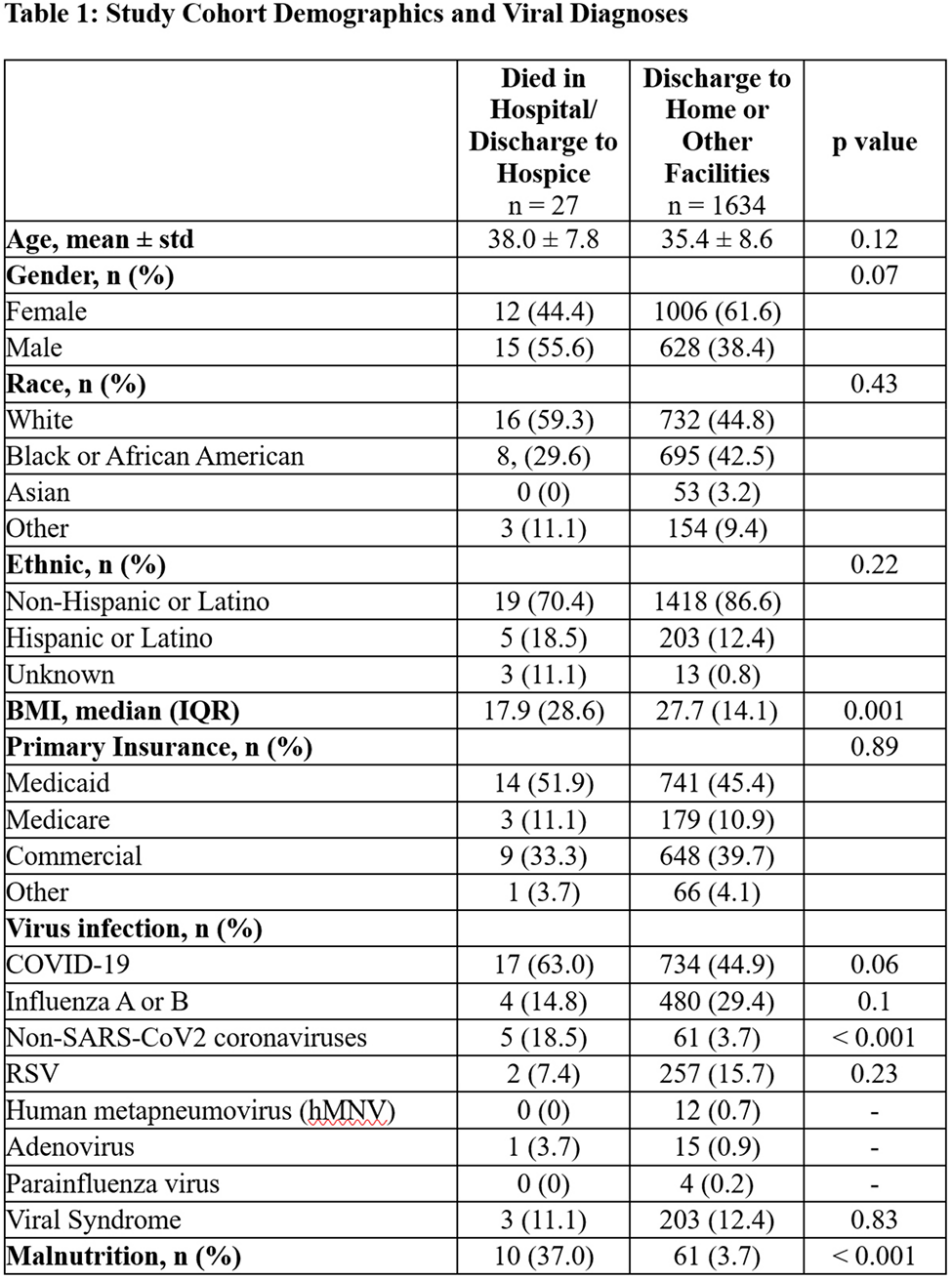

**Figure:**
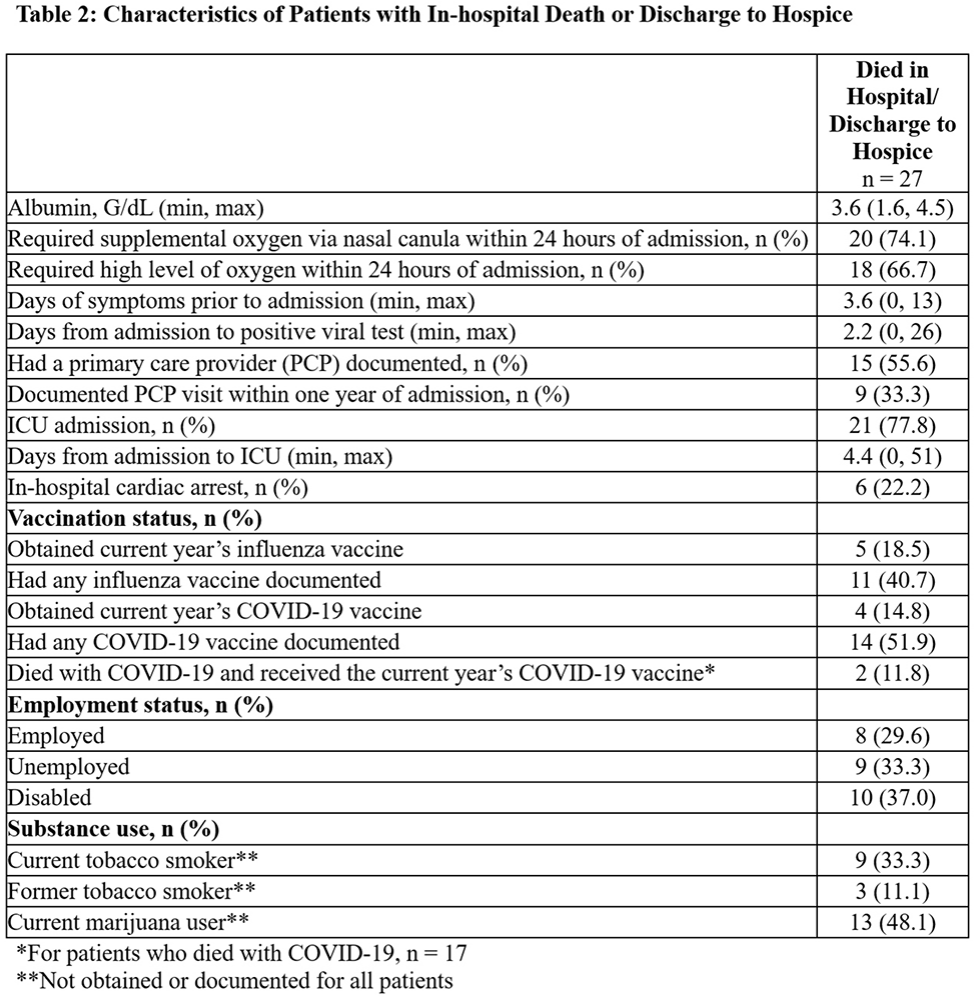

**Methods:**

We conducted a retrospective cohort study at ChristianaCare, a 3-hospital, > 1300-bed community-based academic healthcare system based in Delaware. We included patients hospitalized between Oct 22 - Mar 25, aged 18-50 years, and diagnosed with COVID-19, influenza, RSV, other specific respiratory viruses, or nonspecific viral syndrome based on ICD-10 codes. We obtained demographic and clinical data via the electronic health record and identified comorbidities using Elixhauser/AHRQ comorbidity software redefined for ICD-10-CM. We compared demographic and clinical characteristics of deceased patients to those who survived using chi-square or T-test, and performed chart review for the deceased individuals.

**Results:**

During the 3-year study period, we identified 1661 hospitalized young adults with viral illness, of whom 27 (1.6%) died (19 in-hospital deaths and 8 discharges to hospice). Deceased patients’ demographic characteristics were similar to those who survived, but BMI was much lower and malnutrition more frequent (Table 1). The majority of those who died (63%) were diagnosed with COVID-19. Of the deceased, 9 patients (33.3%) had visited their primary care provider within one year of presentation, 9 (33.3%) were unemployed, and 10 (37%) were disabled (Table 2). Of the 17 patients who died with COVID-19, 2 (11.8%) had received the current year’s vaccine.

**Conclusion:**

Common viruses can be deadly, even in younger populations. Most patients who died, despite having multiple comorbidities, had not seen their primary care provider within one year. The most common viral diagnosis was COVID-19, and few patients were recently vaccinated. These data suggest that COVID-19 remains a significant pathogen, and that vaccination as well as regular primary care may help to reduce mortality.

**Disclosures:**

All Authors: No reported disclosures

